# 8-(Carboxy­methoxy)­quinolinium nitrate monohydrate

**DOI:** 10.1107/S1600536808020357

**Published:** 2008-07-31

**Authors:** Feng Sun, Li Chen, Hua-Cai Fang, Xiao-Ming Lin, Yue-Peng Cai

**Affiliations:** a School of Chemistry and Environment, South China Normal University, Guangzhou 510631, People’s Republic of China

## Abstract

In the title compound, C_11_H_10_NO_3_^+^·NO_3_^−^·H_2_O, the planar 8-carboxy­methoxy­quinolinium cation, the nitrate anion and the water mol­ecule are dimerized by hydrogen bonds into square building-block units, and then further assembled into two-dimensional gently undulating supra­molecular layers.

## Related literature

For general background, see Czugler & Kalman (1981 ▶); Das *et al.* (1987 ▶); Song *et al.* (2004 ▶); Wang & Lu (2004 ▶).
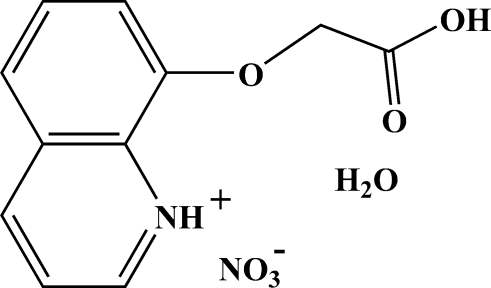


## Experimental

### 

#### Crystal data

C_11_H_10_NO_3_^+^·NO_3_^−^·H_2_O
*M* *_r_* = 284.23
Monoclinic, 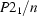

*a* = 5.3577 (5) Å
*b* = 19.5100 (17) Å
*c* = 11.8959 (11) Å
β = 94.663 (3)°
*V* = 1239.3 (2) Å^3^
*Z* = 4
Mo *K*α radiation
μ = 0.13 mm^−1^
*T* = 298 (2) K
0.25 × 0.22 × 0.16 mm

#### Data collection

Bruker SMART CCD area-detector diffractometer
Absorption correction: multi-scan (*SADABS*; Sheldrick, 1996 ▶) *T*_min_ = 0.968, *T*_max_ = 0.980
6235 measured reflections
2071 independent reflections
1376 reflections with *I* > 2σ(*I*)
*R*_int_ = 0.026

#### Refinement

*R*[*F* ^2^ > 2σ(*F* ^2^)] = 0.040
*wR*(*F* ^2^) = 0.107
*S* = 1.01
2071 reflections
189 parameters
3 restraints
H atoms treated by a mixture of independent and constrained refinement
Δρ_max_ = 0.14 e Å^−3^
Δρ_min_ = −0.16 e Å^−3^

### 

Data collection: *SMART* (Bruker, 1998 ▶); cell refinement: *SAINT* (Bruker, 1999 ▶); data reduction: *SAINT*; program(s) used to solve structure: *SHELXS97* (Sheldrick, 2008 ▶); program(s) used to refine structure: *SHELXL97* (Sheldrick, 2008 ▶); molecular graphics: *SHELXTL* (Sheldrick, 2008 ▶); software used to prepare material for publication: *SHELXTL*.

## Supplementary Material

Crystal structure: contains datablocks I, global. DOI: 10.1107/S1600536808020357/rn2043sup1.cif

Structure factors: contains datablocks I. DOI: 10.1107/S1600536808020357/rn2043Isup2.hkl

Additional supplementary materials:  crystallographic information; 3D view; checkCIF report

## Figures and Tables

**Table 1:** Hydrogen-bond geometry (Å, °)

*D*—H⋯*A*	*D*—H	H⋯*A*	*D*⋯*A*	*D*—H⋯*A*
N1—H1⋯O7	0.86	1.81	2.652 (2)	166
O7—H7*B*⋯O5^i^	0.841 (9)	2.610 (18)	3.140 (2)	122.3 (19)
O7—H7*B*⋯O2^ii^	0.841 (9)	2.085 (16)	2.852 (2)	152 (2)
O7—H7*A*⋯O1	0.852 (9)	2.52 (2)	2.943 (2)	111.6 (18)
O7—H7*A*⋯O2	0.852 (9)	1.929 (11)	2.776 (2)	172 (3)
O3—H3⋯O6^iii^	0.82	2.63	3.192 (2)	127
O3—H3⋯N2^iii^	0.82	2.55	3.301 (3)	154
O3—H3⋯O5^iii^	0.82	1.77	2.587 (2)	175

Symmetry codes: (i) 
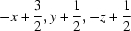
; (ii) 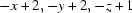; (iii) 
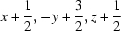
.

**Fig. 1.**
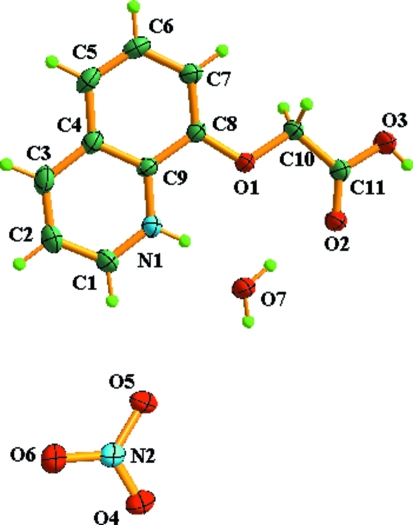
The structure of the title compound (I). The atom-numbering scheme is shown and ellipsoids are drawn at the 30% probability level.

**Fig. 2.**
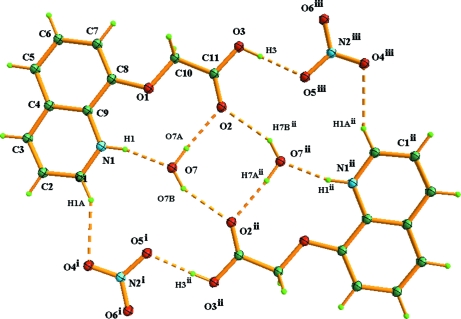
A view of the dimerized supramolecular sheet constructed by hydrogen bonding interactions. Symmetry codes i: 1.5-x, 0.5+y, 0.5-z; ii: 2-x, 2-y, 1-z; iii: 0.5+x, 1.5-y, 0.5+z.

## supplementary crystallographic information

### Comment

There are no reports of the title compound but there are several reports on metal compounds involving the ligand 2-(quinolin-8-yloxy)acetate and its derivatives (Czugler & Kalman, 1981; Das *et al.*, 1987; Wang & Lu, 2004; Song *et al.*, 2004). The title compound (Scheme I) contains one cation, one nitrate anion and one water molecule (Fig.1).

Fig. 2 shows that the cation, the nitrate anion and water molecule are dimerized by the hydrogen bonds (Table 1) into stable square building block units, and then further assembled into 2D supramolecular layers which are gently undulating.

### Experimental

A solution of Cu(NO_3_)_2_ (363 mg, 1.00 mmol) in CH_3_OH (20 ml) was slowly added to a solution of 2-(quinolin-8-yloxy)acetic acid (410 mg, 1.95 mmol) in CH_3_OH (10 ml). The resultant blue solution was stirred for 2 h at room temperature and then filtered. Colorless crystals suitable for X-ray diffraction were obtained in two day by slow diffusion of diethyl ether into a dilute solution of the title complex in methanol. The assigned structure was substantiated by elemental analysis; calculated for C_11_H_12_N_2_O_7_: C 46.44, H 4.22, N 9.85%; found: C 46.36, H 4.28, N 9.82%.

### Refinement

The structure was solved using direct methods followed by Fourier synthesis. Non-H atoms were refined anisotropically. All of H atoms except water molecule were placed in idealized positions (C—H = 0.93 or 0.97 Å, O—H = 0.82 Å, N—H = 0.86 Å), forced to ride on the atom to which they are bonded, and were included in the refinement in the riding-model approximation. U_iso_ values were set equal to 1.5U_eq_(parent atom) for methyl H atoms and to 1.2U_eq_(parent atom)for all other H atoms. The water H atoms were located in a difference Fourier map, and were refined with distance restraints of O—H = 0.84 (1) and H···H 1.43 (2) Å, but their U_iso_ values were set equal to 1.5 U_eq_(parent atom O).

### Crystal data

**Table:**

C_11_H_10_NO_3_^+^·NO_3_^–^·H_2_O	*F*_000_ = 592
*M**_r_* = 284.23	*D*_x_ = 1.523 Mg m^−^^3^
Monoclinic, *P*2_1_/*n*	Mo *K*α radiation λ = 0.71073 Å
Hall symbol: -P2yn	Cell parameters from 1544 reflections
*a* = 5.3577 (5) Å	θ = 2.7–22.5º
*b* = 19.5100 (17) Å	µ = 0.13 mm^−^^1^
*c* = 11.8959 (11) Å	*T* = 298 (2) K
β = 94.663 (3)º	Block, yellow
*V* = 1239.3 (2) Å^3^	0.25 × 0.22 × 0.16 mm
*Z* = 4	

### Data collection

**Table:**

Bruker SMART CCD area-detector diffractometer	2071 independent reflections
Radiation source: fine-focus sealed tube	1376 reflections with *I* > 2σ(*I*)
Monochromator: graphite	*R*_int_ = 0.026
*T* = 298(2) K	θ_max_ = 25.5º
φ and ω scans	θ_min_ = 2.0º
Absorption correction: multi-scan(SADABS; Sheldrick, 1996)	*h* = −6→6
*T*_min_ = 0.968, *T*_max_ = 0.980	*k* = −23→22
6235 measured reflections	*l* = −14→13

### Refinement

**Table:**

Refinement on *F*^2^	Secondary atom site location: difference Fourier map
Least-squares matrix: full	Hydrogen site location: inferred from neighbouring sites
*R*[*F*^2^ > 2σ(*F*^2^)] = 0.040	H atoms treated by a mixture of independent and constrained refinement
*wR*(*F*^2^) = 0.107	*w* = 1/[σ^2^(*F*_o_^2^) + (0.0466*P*)^2^ + 0.237*P*] where *P* = (*F*_o_^2^ + 2*F*_c_^2^)/3
*S* = 1.02	(Δ/σ)_max_ = 0.001
2071 reflections	Δρ_max_ = 0.14 e Å^−^^3^
189 parameters	Δρ_min_ = −0.16 e Å^−^^3^
3 restraints	Extinction correction: SHELXL97 (Sheldrick, 2008), Fc^*^=kFc[1+0.001xFc^2^λ^3^/sin(2θ)]^-1/4^
Primary atom site location: structure-invariant direct methods	Extinction coefficient: 0.0044 (12)

### Special details

**Table:**

Geometry. All e.s.d.'s (except the e.s.d. in the dihedral angle between two l.s. planes) are estimated using the full covariance matrix. The cell e.s.d.'s are taken into account individually in the estimation of e.s.d.'s in distances, angles and torsion angles; correlations between e.s.d.'s in cell parameters are only used when they are defined by crystal symmetry. An approximate (isotropic) treatment of cell e.s.d.'s is used for estimating e.s.d.'s involving l.s. planes.
Refinement. Refinement of *F*^2^ against ALL reflections. The weighted *R*-factor *wR* and goodness of fit *S* are based on *F*^2^, conventional *R*-factors *R* are based on *F*, with *F* set to zero for negative *F*^2^. The threshold expression of *F*^2^ > σ(*F*^2^) is used only for calculating *R*-factors(gt) *etc*. and is not relevant to the choice of reflections for refinement. *R*-factors based on *F*^2^ are statistically about twice as large as those based on *F*, and *R*- factors based on ALL data will be even larger.

### Fractional atomic coordinates and isotropic or equivalent isotropic displacement parameters (Å^2^)

**Table:**

	*x*	*y*	*z*	*U*_iso_*/*U*_eq_	
O1	0.3857 (3)	1.04106 (6)	0.31886 (12)	0.0504 (4)	
O2	0.7769 (3)	1.08262 (7)	0.45561 (14)	0.0639 (5)	
O3	0.6421 (3)	1.19011 (7)	0.44971 (15)	0.0717 (5)	
H3	0.7765	1.1975	0.4856	0.107*	
O4	0.7649 (4)	0.21455 (8)	0.17156 (19)	0.0945 (7)	
O5	0.5562 (3)	0.28962 (8)	0.07269 (16)	0.0761 (6)	
O6	0.4361 (4)	0.18475 (9)	0.07206 (17)	0.0943 (7)	
O7	0.7613 (3)	0.94258 (8)	0.41435 (15)	0.0697 (5)	
H7A	0.766 (4)	0.9861 (5)	0.420 (2)	0.105*	
H7B	0.893 (3)	0.9224 (10)	0.439 (2)	0.105*	
N1	0.3815 (3)	0.90790 (8)	0.26857 (14)	0.0478 (5)	
H1	0.4942	0.9259	0.3150	0.057*	
N2	0.5863 (4)	0.22855 (10)	0.10544 (17)	0.0595 (5)	
C1	0.3960 (5)	0.84190 (10)	0.24671 (19)	0.0585 (6)	
H1A	0.5251	0.8159	0.2820	0.070*	
C2	0.2210 (5)	0.81128 (12)	0.1718 (2)	0.0678 (7)	
H2	0.2335	0.7649	0.1552	0.081*	
C3	0.0298 (5)	0.84926 (12)	0.1222 (2)	0.0633 (7)	
H3A	−0.0890	0.8285	0.0719	0.076*	
C4	0.0098 (4)	0.91954 (11)	0.14604 (17)	0.0491 (6)	
C5	−0.1837 (5)	0.96193 (13)	0.09874 (19)	0.0597 (6)	
H5	−0.3112	0.9434	0.0503	0.072*	
C6	−0.1842 (5)	1.02966 (12)	0.12375 (19)	0.0592 (6)	
H6	−0.3134	1.0571	0.0923	0.071*	
C7	0.0059 (4)	1.05930 (11)	0.19611 (18)	0.0503 (6)	
H7	0.0039	1.1062	0.2103	0.060*	
C8	0.1930 (4)	1.01977 (9)	0.24550 (16)	0.0428 (5)	
C9	0.1954 (4)	0.94906 (10)	0.22072 (16)	0.0415 (5)	
C10	0.4012 (4)	1.11253 (9)	0.34252 (17)	0.0480 (6)	
H10A	0.2518	1.1275	0.3764	0.058*	
H10B	0.4137	1.1380	0.2732	0.058*	
C11	0.6279 (4)	1.12555 (11)	0.42193 (17)	0.0482 (6)	

### Atomic displacement parameters (Å^2^)

**Table:**

	*U*^11^	*U*^22^	*U*^33^	*U*^12^	*U*^13^	*U*^23^
O1	0.0499 (10)	0.0361 (7)	0.0612 (9)	0.0015 (7)	−0.0185 (8)	−0.0028 (6)
O2	0.0534 (11)	0.0450 (9)	0.0881 (11)	0.0037 (8)	−0.0256 (9)	−0.0097 (8)
O3	0.0775 (14)	0.0385 (8)	0.0930 (13)	−0.0001 (8)	−0.0295 (10)	−0.0109 (8)
O4	0.0822 (14)	0.0620 (11)	0.1291 (16)	0.0015 (10)	−0.0530 (13)	0.0185 (11)
O5	0.0753 (13)	0.0433 (9)	0.1048 (13)	0.0006 (8)	−0.0219 (10)	0.0184 (9)
O6	0.0987 (16)	0.0594 (10)	0.1156 (15)	−0.0244 (11)	−0.0464 (13)	0.0157 (10)
O7	0.0638 (12)	0.0514 (9)	0.0877 (12)	0.0058 (8)	−0.0320 (10)	−0.0014 (9)
N1	0.0471 (12)	0.0411 (10)	0.0533 (10)	−0.0052 (9)	−0.0074 (9)	0.0001 (8)
N2	0.0587 (14)	0.0471 (12)	0.0697 (13)	0.0000 (11)	−0.0124 (11)	0.0048 (10)
C1	0.0633 (17)	0.0381 (12)	0.0723 (15)	−0.0001 (11)	−0.0047 (14)	0.0011 (11)
C2	0.077 (2)	0.0438 (13)	0.0808 (17)	−0.0116 (13)	−0.0052 (15)	−0.0090 (12)
C3	0.0669 (18)	0.0577 (14)	0.0630 (15)	−0.0201 (13)	−0.0083 (14)	−0.0099 (12)
C4	0.0439 (14)	0.0542 (13)	0.0474 (12)	−0.0082 (11)	−0.0059 (11)	−0.0006 (10)
C5	0.0461 (16)	0.0749 (17)	0.0552 (14)	−0.0107 (13)	−0.0143 (12)	−0.0008 (12)
C6	0.0450 (15)	0.0719 (16)	0.0579 (14)	0.0072 (13)	−0.0120 (12)	0.0073 (12)
C7	0.0455 (15)	0.0474 (12)	0.0562 (13)	0.0041 (11)	−0.0061 (12)	0.0045 (10)
C8	0.0405 (14)	0.0433 (12)	0.0429 (11)	−0.0027 (10)	−0.0068 (11)	0.0010 (9)
C9	0.0361 (13)	0.0434 (11)	0.0437 (11)	−0.0018 (10)	−0.0038 (10)	0.0036 (9)
C10	0.0508 (15)	0.0347 (11)	0.0560 (13)	0.0005 (10)	−0.0102 (11)	0.0011 (9)
C11	0.0506 (15)	0.0400 (12)	0.0528 (13)	−0.0033 (11)	−0.0038 (11)	−0.0008 (10)

### Geometric parameters (Å, °)

**Table:**

O1—C8	1.362 (2)	C2—H2	0.9300
O1—C10	1.424 (2)	C3—C4	1.406 (3)
O2—C11	1.203 (2)	C3—H3A	0.9300
O3—C11	1.303 (2)	C4—C9	1.402 (3)
O3—H3	0.8200	C4—C5	1.407 (3)
O4—N2	1.219 (2)	C5—C6	1.355 (3)
O5—N2	1.260 (2)	C5—H5	0.9300
O6—N2	1.218 (2)	C6—C7	1.403 (3)
O7—H7A	0.852 (9)	C6—H6	0.9300
O7—H7B	0.841 (9)	C7—C8	1.360 (3)
N1—C1	1.317 (2)	C7—H7	0.9300
N1—C9	1.368 (2)	C8—C9	1.411 (3)
N1—H1	0.8600	C10—C11	1.498 (3)
C1—C2	1.377 (3)	C10—H10A	0.9700
C1—H1A	0.9300	C10—H10B	0.9700
C2—C3	1.359 (3)		
			
C8—O1—C10	117.04 (15)	C4—C5—H5	120.0
C11—O3—H3	109.5	C5—C6—C7	121.5 (2)
H7A—O7—H7B	114.9 (13)	C5—C6—H6	119.2
C1—N1—C9	122.98 (19)	C7—C6—H6	119.2
C1—N1—H1	118.5	C8—C7—C6	120.3 (2)
C9—N1—H1	118.5	C8—C7—H7	119.9
O6—N2—O4	121.1 (2)	C6—C7—H7	119.9
O6—N2—O5	119.9 (2)	C7—C8—O1	126.71 (18)
O4—N2—O5	119.1 (2)	C7—C8—C9	118.90 (19)
N1—C1—C2	120.3 (2)	O1—C8—C9	114.39 (17)
N1—C1—H1A	119.9	N1—C9—C4	118.56 (18)
C2—C1—H1A	119.9	N1—C9—C8	120.38 (18)
C3—C2—C1	119.6 (2)	C4—C9—C8	121.06 (19)
C3—C2—H2	120.2	O1—C10—C11	108.78 (16)
C1—C2—H2	120.2	O1—C10—H10A	109.9
C2—C3—C4	120.7 (2)	C11—C10—H10A	109.9
C2—C3—H3A	119.6	O1—C10—H10B	109.9
C4—C3—H3A	119.6	C11—C10—H10B	109.9
C9—C4—C3	117.8 (2)	H10A—C10—H10B	108.3
C9—C4—C5	118.3 (2)	O2—C11—O3	124.4 (2)
C3—C4—C5	123.9 (2)	O2—C11—C10	125.03 (19)
C6—C5—C4	120.0 (2)	O3—C11—C10	110.57 (19)
C6—C5—H5	120.0		
			
C9—N1—C1—C2	0.8 (3)	C1—N1—C9—C4	0.8 (3)
N1—C1—C2—C3	−1.4 (4)	C1—N1—C9—C8	−178.5 (2)
C1—C2—C3—C4	0.4 (4)	C3—C4—C9—N1	−1.7 (3)
C2—C3—C4—C9	1.1 (3)	C5—C4—C9—N1	178.85 (19)
C2—C3—C4—C5	−179.4 (2)	C3—C4—C9—C8	177.63 (19)
C9—C4—C5—C6	1.5 (3)	C5—C4—C9—C8	−1.8 (3)
C3—C4—C5—C6	−177.9 (2)	C7—C8—C9—N1	179.70 (19)
C4—C5—C6—C7	0.3 (4)	O1—C8—C9—N1	−0.3 (3)
C5—C6—C7—C8	−1.8 (3)	C7—C8—C9—C4	0.4 (3)
C6—C7—C8—O1	−178.6 (2)	O1—C8—C9—C4	−179.64 (18)
C6—C7—C8—C9	1.4 (3)	C8—O1—C10—C11	−178.52 (17)
C10—O1—C8—C7	−3.3 (3)	O1—C10—C11—O2	3.0 (3)
C10—O1—C8—C9	176.72 (17)	O1—C10—C11—O3	−177.08 (18)

### Hydrogen-bond geometry (Å, °)

**Table:**

*D*—H···*A*	*D*—H	H···*A*	*D*···*A*	*D*—H···*A*
N1—H1···O7	0.86	1.81	2.652 (2)	166
O7—H7B···O5^i^	0.841 (9)	2.610 (18)	3.140 (2)	122.3 (19)
O7—H7B···O2^ii^	0.841 (9)	2.085 (16)	2.852 (2)	152 (2)
O7—H7A···O1	0.852 (9)	2.52 (2)	2.943 (2)	111.6 (18)
O7—H7A···O2	0.852 (9)	1.929 (11)	2.776 (2)	172 (3)
O3—H3···O6^iii^	0.82	2.63	3.192 (2)	127
O3—H3···N2^iii^	0.82	2.55	3.301 (3)	154
O3—H3···O5^iii^	0.82	1.77	2.587 (2)	175

Symmetry codes: (i) −*x*+3/2, *y*+1/2, −*z*+1/2; (ii) −*x*+2, −*y*+2, −*z*+1; (iii) *x*+1/2, −*y*+3/2, *z*+1/2.

## References

[bb1] Bruker (1998). *SMART* Bruker AXS Inc., Madison, Wisconsin, USA.

[bb2] Bruker (1999). *SAINT* Bruker AXS Inc., Madison, Wisconsin, USA.

[bb3] Czugler, M. & Kalman, A. (1981). *J. Mol. Struct.* **75**, 29–37.

[bb4] Das, V. G. K., Wei, C., Ng, S. W. & Mak, T. C. W. (1987). *J. Organomet. Chem.* **322**, 33–47.

[bb5] Sheldrick, G. M. (1996). *SADABS* University of Göttingen, Germany.

[bb6] Sheldrick, G. M. (2008). *Acta Cryst.* A**64**, 112–122. 10.1107/S0108767307043930 18156677

[bb7] Song, R.-F., Wang, Y.-H. & Jiang, F. (2004). *Acta Cryst.* E**60**, m1695–m1696.

[bb8] Wang, Y.-H. & Lu, F. (2004). *Acta Cryst.* C**60**, m557–m559. 10.1107/S0108270104022048 15528802

